# Revision and Validation of the Hwa-Byung Scale

**DOI:** 10.1192/j.eurpsy.2022.1393

**Published:** 2022-09-01

**Authors:** J. Kim, S.-A. Park, S. Lee, Y. Kwan, S.-W. Choi

**Affiliations:** 1 Duksung Women’s University, Clinical Psychology Lab, Dobong-gu, Seoul, Republic of Korea; 2 Yonsei University Wonju College of Medicine, Psychiatry, Wonju, Korea, Republic of

**Keywords:** Hwa-Byung, scale revision, Anger, cultural-related syndrome

## Abstract

**Introduction:**

Hwa-Byung is a cultural-related mental syndrome that reflects the cultural characteristics of Korean in DSM-IV. This syndrome is caused by anger or resentment towards unreasonable social violence and trauma.

**Objectives:**

The purpose of this study is to revise and validate the ’Hwa-Byung scale’, which can be used to diagnose Hwa-Byung and evaluate the severity of its symptoms.

**Methods:**

To begin with, the factors of the Hwa-Byung scale were set based on the previous studies. Additionally, the respective subfactors were generated by the semi-structured interviews with these patients. Based on these factors and previous studies, a 142-item pool was developed and verified by six Oriental Neuropsychiatrists. A pilot study was conducted on 50 patients with Hwa-Byung and the main study for the validation was conducted on 200 Hwa-Byung patients. Item analysis, internal consistency, and exploratory/confirmatory factor analysis were performed. Lastly, this study analyzed the ROC curve to present the diagnostic cut-off score of the scale.

**Results:**

As a result of analyzing the content validity of the item pool, we constructed a preliminary scale. We excluded the inadequate questions from the pilot study results. In the main study, The Hwa-Byung scale showed high internal consistency and its items were suitable for the factor structure. Finally, we suggest an optimal cut-off score of the symptoms sub-scale for screening Hwa-Byung.

**Conclusions:**

Overall, the results of this study indicated the reliability and validity of the Hwa-Byung Scale. Based on these results, we discussed several values and limitations of this study and provided suggestions for further research.

**Disclosure:**

No significant relationships.

